# Preoperative Differentiation of Benign and Malignant Non-epithelial Ovarian Tumors: Clinical Features and Tumor Markers

**DOI:** 10.1055/s-0040-1712993

**Published:** 2020-09-29

**Authors:** Tiago Augusto Gomes, Elizabeth Aparecida Campos, Adriana Yoshida, Luís Otavio Sarian, Liliana Aparecida Lucci de Angelo Andrade, Sophie Françoise Derchain

**Affiliations:** 1 Department of Obstetrics and Gynecology, Faculty of Medical Sciences, Universidade Estadual de Campinas, Campinas, São Paulo, SP, Brazil; 2 Laboratory of Experimental Pathology, Hospital da Mulher Prof. Dr. José Aristodemo Pinotti, Universidade Estadual de Campinas, Campinas, São Paulo, Brazil; 3 Department of Pathology, Faculty of Medical Sciences, universidade Estadual de Campinas, Campinas, SP, Brazil

**Keywords:** non-epithelial ovarian tumors, ovarian cancer, biomarkers, germ cell tumors, sex cord-stromal tumors, tumores ovarianos não epiteliais, câncer de ovário, biomarcadores, tumores de células germinativas, tumores do cordão sexual-estroma

## Abstract

**Objective** To evaluate the role of clinical features and preoperative measurement of cancer antigen 125 (CA125), human epididymis protein (HE4), and carcinoembryonic antigen (CEA) serum levels in women with benign and malignant non-epithelial ovarian tumors.

**Methods** One hundred and nineteen consecutive women with germ cell, sex cord-stromal, and ovarian leiomyomas were included in this study. The preoperative levels of biomarkers were measured, and then surgery and histopathological analysis were performed. Information about the treatment and disease recurrence were obtained from the medical files of patients.

**Results** Our sample included 71 women with germ cell tumors (64 benign and 7 malignant), 46 with sex cord-stromal tumors (32 benign and 14 malignant), and 2 with ovarian leiomyomas. Among benign germ cell tumors, 63 were mature teratomas, and, among malignant, four were immature teratomas. The most common tumors in the sex cord-stromal group were fibromas (benign) and granulosa cell tumor (malignant). The biomarker serum levels were not different among benign and malignant non-epithelial ovarian tumors. Fertility-sparing surgeries were performed in 5 (71.4%) women with malignant germ cell tumor. Eleven (78.6%) patients with malignant sex cord-stromal tumors were treated with fertility-sparing surgeries. Five women (71.4%) with germ cell tumors and only 1 (7.1%) with sex cord-stromal tumor were treated with chemotherapy. One woman with germ cell tumor recurred and died of the disease and one woman with sex cord-stromal tumor recurred.

**Conclusion** Non-epithelial ovarian tumors were benign in the majority of cases, and the malignant cases were diagnosed at initial stages with good prognosis. The measurements of CA125, HE4, and CEA serum levels were not useful in the preoperative diagnosis of these tumors.

## Resumo

**Objetivo** Avaliar o papel das características clínicas e a medida pré-operatória dos níveis séricos de CA125, HE4, e CEA em mulheres com tumores de ovário não epiteliais benignos e malignos.

**Métodos** Cento e dezenove mulheres consecutivas com tumores ovarianos de células germinativas, do cordão sexual-estroma, e miomas ovarianos foram incluídas neste estudo. Os níveis pré-operatórios dos biomarcadores foram medidos, a cirurgia e a análise histopatológica foram realizadas. Informações sobre tratamento e recorrência da doença foram obtidas dos prontuários médicos das pacientes.

**Resultados** Nossa amostra incluiu 71 mulheres com tumores de células germinativas (64 benignos e 7 malignos), 46 com tumores do cordão sexual-estroma (32 benignos e 14 malignos), e 2 com leiomiomas ovarianos. Entre os tumores benignos de células germinativas, 63 eram teratomas maduros, e, entre os malignos, quatro eram teratomas imaturos. Os tumores mais comuns do grupo do cordão sexual-estroma foram fibromas (benignos) e tumores de células da granulosa (malignos). Os níveis séricos dos biomarcadores não diferiram entre os tumores de ovário não epiteliais benignos e malignos. A cirurgia preservadora de fertilidade foi realizada em 5 (71,4%) mulheres com tumores malignos de células germinativas. Onze (78,6%) mulheres com tumores do cordão sexual-estroma malignos foram tratadas com cirurgia preservadora de fertilidade. Cinco (71,4%) mulheres com células germinativas e apenas 1 (7,1%) com tumor do cordão sexual-estroma foram tratadas com quimioterapia. Uma mulher com tumor de células germinativas recidivou e morreu da doença. Uma mulher com tumor do cordão sexual-estroma recidivou.

**Conclusão** Os tumores de ovário não epiteliais foram benignos na maioria dos casos e os malignos foram diagnosticados em estágios iniciais, com bom prognóstico. A medida dos níveis séricos de CA125, HE4, e CEA não foram úteis no diagnóstico pré-operatório desses tumores.

## Introduction

Adnexal masses are commonly found on gynecological imaging in women of all ages. 1 It is estimated that 5 to 10% of women will be submitted to surgery to investigate an adnexal mass in their lifetime. 2 Despite the majority of them being benign, malignant tumors must be promptly diagnosed and treated. 3 Non-epithelial ovarian cancers are rare, accounting for approximately only 5% of ovarian malignancies encompassing germ cell tumors (3%) and sex cord-stromal tumors (2%). 4

According to the last classification of World Health Organization (WHO), 5 germ cell tumors of ovary comprise dysgerminoma, yolk sac tumor, embryonal carcinoma, non-gestational choriocarcinoma, mature teratoma, immature teratoma, mixed germ cell tumor, monodermal teratomas and tumors with malignant transformation arising from a dermoid cyst. Mature teratoma, is the most common benign ovarian neoplasia, most occur during reproductive years, with a peak incidence between 20 and 40 years of age. 6 Benign mature teratomas comprise 95% of all germ cell tumors, and only 5% of germ cell tumors are malignant. 6

The WHO last classification for ovarian sex cord-stromal tumors shows that these tumors have been reclassified into the following clinicopathologic entities: pure stromal tumors, pure sex cord tumors, and mixed sex cord-stromal tumors. Ovarian fibroma, thecoma, Leydig cell tumor are pure stromal tumors. Adult granulosa cell tumor, Juvenile granulosa cell tumor and Sertoli cell tumor are pure sex cord tumors, while Sertoli-Leydig cell tumor is mixed sex cord-stromal tumor. 5 7

In clinical practice, women undergo surgery to diagnose and treat an adnexal mass according to findings on clinical exam, transvaginal ultrasound and tumor biomarker. More specific biomarkers such as β-subunit of human chorionic gonadotropin (β-hCG), α fetoprotein, lactic dehydrogenase (LDH) may be useful for diagnosing malignant non-epithelial tumors. 8 On the other hand, there is scarce information in the current literature about the role of routinely measured biomarkers cancer antigen 125 (CA125) and carcinoembryonic antigen (CEA); and also more recently discovered biomarker Human Epididymis Protein 4 (HE4) in the preoperative diagnosis of non-epithelial ovarian tumors.

The objective of our study was to evaluate, the role of clinical features and preoperative determination of CA125, HE4 and CEA serum levels in the differentiation of benign from malignant non-epithelial ovarian tumors.

## Methods

### Patients

This is a cross-sectional study that was conducted at Hospital da Mulher Prof. Dr. José Aristodemo Pinotti at Universidade Estadual de Campinas (Unicamp), from February 2010 to December 2015. The study and was approved by the research ethics committee of the institution (Protocol 1092/2009). Women referred to the pelvic oncology clinic, due to adnexal masses detected in ultrasound or other imaging exam, were invited to participate. Patients were consecutively included after signing a consent form and were submitted to the study protocol. On the first visit, patients were submitted to physical exam, and blood was collected to measure biomarker levels. In addition, transvaginal ultrasound was scheduled. When indicated, women underwent surgery for disease diagnosis and treatment. The indication of surgery was based on clinical exam, preoperative biomarkers, and ultrasound scan. The medical files of the patients obtained, which were from the hospital's digital filing system, were reviewed to obtain information about treatment, disease recurrence, and patient status. Women were considered postmenopausal when they had > 1 year of amenorrhea or were > 50 years old in case of previous hysterectomy. In premenopausal women, tumorectomy or unilateral adnexectomy with contralateral ovarian preservation without hysterectomy was considered fertility-sparing surgery.

### Ultrasound (US)

The following ultrasound parameters were used to decide which women should undergo surgical treatment: largest diameter of the lesion; maximum diameter of the largest solid part; if unilocular or multilocular; presence of wall irregularity; ascites; acoustic shadows; number of papillary projections; color Doppler blood flow. 9 When surgery was not indicated, women were scheduled for clinical follow-up. From 869 women enrolled in the study, we excluded 361 who were not operated, 237 women with epithelial ovarian tumors, 128 with non-neoplastic and non-ovarian tumors, and 24 with ovarian metastases. One hundred and nineteen consecutive women with benign and malignant non-epithelial ovarian tumors were included in the study. Women had their surgery indicated according to their clinical exam, ultrasound results (simple rules by international ovarian tumor analysis [IOTA]), 9 and serum biomarkers. In the present study, 72 women had their ultrasound analyzed with IOTA simple rules. Among them, 9 had malignant tumors as per histology diagnosis, but the IOTA simple rules were malignant in 8 cases and benign in 1 case of granulosa cell tumor. Among the remaining 63 cases that had a benign tumor histology diagnosis, the IOTA simple rules were benign in 54 women, indeterminate in 1 case (ovarian fibroma), and malignant in 8 women (2 with fibroma, 1 with thecoma, 1 with ovarian leiomyoma, 1 with struma ovarii, and 3 with teratomas).

### Histopathology

Histopathological diagnosis was the gold standard parameter, performed by pathologists specialized in gynecologic pathology, following the last “WHO classification of tumors of female reproductive organs.” 5 Women with bilateral tumors with one of them presenting epithelial histology were excluded from the study. Our sample comprises 71 women with germ cell tumors (64 benign and 7 malignant), 46 with sex cord-stromal tumors (32 benign and 14 malignant), and 2 with ovarian leiomyomas.

### CA125 and CEA

Serum levels of CA125 and CEA were determined by the CA125 II and CEA tests, respectively, both biomarkers through the chemiluminescence technic in the automatic analyzer Cobas e411 (Roche Diagnostics GmbH, Mannheim, Germany) according to the manufacturer's instructions, with CA125 expressed in U/ml and CEA in ng/ml.

### Human Epididymis Protein 4 (HE4)

The concentrations of HE4 were measured according to manufacturer's instructions using the ARCHITECT HE4 assay (Abbott Diagnostics, Abbott Park, IL, USA), with HE4 expressed in pmol/L.

### Statistical Analysis

Data were analyzed using the R Environment for Statistical Computing software (R Foundation for Statistical Computing, Vienna, Austria). 10 According to the histopathological diagnoses, tumors were classified as benign or malignant germ cell or sex cord-stromal tumors. Two patients with ovarian leiomyoma had their tumors grouped with sex cord-stromal tumors for statistical purposes. Women's clinical features, serum biomarker levels and surgical treatment were compared using the Chi-square test for categorical variables and the Kruskal-Wallis test for the continuous variables. Statistical calculations were performed using 95% confidence intervals (95% CIs) considering *p* < 0.05 as significant. For missing data reason, we excluded 19 cases without CA125, 18 cases without HE4, and 4 without CEA for the biomarkers' analysis only. Follow-up time (in months) was considered from the date of diagnostic surgery to last hospital visit or date of death in the case of one patient.

## Results

In Table 1 , among 119 women, 98 (82.4%) presented benign tumors and 21 (17.6%) malignant. Seventy-two (60.5%) were premenopausal and 47 (39.5%) were postmenopausal. Germ cell tumors were significantly more frequent in premenopausal women, and those younger than 50 years old, compared with women with sex cord-stromal tumors. Neither body mass index nor history of close relatives with breast or ovarian cancer were related to tumor malignancy. Benign ovarian tumors were bilateral in 16 (16.3% of benign tumors) women. Almost all women with malignant germ cell and sex cord-stromal tumors presented at the initial stage of the disease: 20 (95.2%) with stage I, of note, 15 (71.4%) were stage Ia. Among benign germ cell tumors, 63 (98.4%) were mature teratomas, and, among malignant, only 4 (57.1%) were immature teratomas. Fibromas were the most common tumors among the patients in the benign sex cord-stromal group, and in the malignant counterpart, the most common was granulosa cell tumor.

**Table 1:** Clinical features by tumor type

Characteristics	Germ cell tumors ( *n* = 71)			Sex cord-stromal tumors ( *n* = 48)			
	Benign n = 64 (%)	Malignant n = 7 (%)	P1	Benign n = 34 (%)	Malignant n = 14 (%)	P2	P3
**Age (years)**							
**< 35**	29 (45.3)	6 (85.7)		4 (11.8)	4 (28.6)		
**35–50**	17 (26.6)	0	0.110	6 (17.6)	5 (35.7)	0.125	0.0001
**> 50**	18 (28.1)	1 (14.3)		24 (70.6)	5 (35.7)		
**Menopausal status**							
**Premenopausal**	48 (75)	6 (85.7)	0.528	9 (26.5)	9 (64.3)	0.024	0.0001
**Postmenopausal**	16 (25)	1 (14.3)		25 (73.5)	5 (35.7)		
** Body mass index (kg/m ^2^ ) **							
**< 30**	44 (68.7)	4 (57.1)		23 (67.7)	8 (57.1)		
**30–35**	14 (21.9)	2 (28.6)	0.816	8 (23.5)	4 (28.6)	0.842	0.963
**> 35**	6 (9.4)	1 (14.3)		3 (8.8)	2 (14.3)		
**Close relatives with cancer (breast/ovary)**							
**No**	31 (48.4)	2 (28.6)	0.437	16 (47.1)	9 (64.3)	0.232	0.478
**Yes**	33 (51.6)	5 (71.4)		18 (52.9)	5 (35.7)		
**Laterality**							
**Unilateral**	53 (82.8)	6 (85.7)	0.845	29 (85.3)	14 (100)	0.117	0.301
**Bilateral**	11(17.2)	1 (14.3)		5 (14.7)	0		
**Stage**							
**I**		7 (100)			13 (92.9)		
**II**							
**III**					1 (7.1)		
**IV**							
**Histological subtype**							
**Mature teratoma**	63 (98.4)						
**Struma ovarii**	1 (1.6)						
**Immature teratoma**		4 (57.1)					
**Carcinoid**		1 (14.3)					
**Dysgerminoma**		1 (14.3)					
**Yolk sac tumor**		1 (14.3)					
**Fibroma**				26 (76.5)			
**Thecoma**				5 (14.7)			
**Ovarian leiomyoma**				2 (5.9)			
**Sclerosing stromal tumor**				1 (2.9)			
**Granulosa cell**					10 (71.4)		
**Steroid cell tumor**					1 (7.1)		
**Sertoli-Leydig cell tumor**					2 (14.3)		
Gynandroblastoma *					1 (7.1)		

Abbreviations: P1, including only germ cell tumors; P2, including only sex cord-stromal tumors; P3, including germ cell and sex cord-stromal tumors.

* currently known as sex cord-stromal tumor, not otherwise specified; which is a subtype of the mixed sex cord-stromal tumors group.

In table 2 , mean CA125 and HE4 were higher in malignant germ cell tumors, although without statistical significance. Among them, a postmenopausal patient with carcinoid tumor presented an HE4 value of 211.2 pmol/l. There was no difference in the expression of these markers in women with benign or malignant sex cord-stromal tumors, regarding mean serum levels. The analysis of biomarkers concentration by cutoff points showed that women with malignant germ cell tumors presented significantly elevated CA125, HE4, and CEA levels.

**Table 2:** Mean serum levels of CA125, HE4, and CEA and distribution by cutoff points according to histopathological diagnosis

Biomarkers	Germ cell tumor *n* = 71			Sex cord-stromal tumors *n* = 48		
	Benign n = 64	Malignant n = 7	*p* -value	Benign n = 34	Malignant n = 14	*p* -value
**CA 125 (U/ml)**	63.8	376	0.37	90.9	65.8	0.53
	(11–29.3)	(89.8–518)		(13–33.9)	(9.2–63.3)	
**HE4 (pmol/L)**	46.5	114.0	0.44	64.3	53.5	0.47
	(33.9–54.6)	(67.5–167.5)		(47.3–78.8)	(45.1–59.5)	
**CEA (ng/ml)**	4.4	3.9	0.46	2.6	1.8	0.49
	(1.2–2.9)	(1.6–5.8)		(1.2–3.0)	(1.1–2.6)	
**Biomarkers**						
**CA 125**						
**< 35 (U/ml)**	46	2	0.0002	22	7	0.1889
**≥ 35 (U/ml)**	7	5		6	5	
**HE4**						
**Premenopausal**						
**< 70 (pmol/L)**	39	2	0.00002	5	8	0.3747
**≥ 70 (pmol/L)**	1	4		2	1	
**Postmenopausal**						
**< 140 (pmol/L)**	13	0	0.0001	20	5	0.6187
**≥ 140 (pmol/L)**	0	1		1	0	
**CEA**						
**< 5 (ng/ml)**	47	4	0.028	28	14	0.1662
**≥ 5 (ng/ml)**	6	3		4	0	

Abbreviations: CA125, cancer antigen 125; CEA, carcinoembryonic antigen; HE4, human epididymis protein.

Missing data - CA125 for 19 women, HE4 for 18 women and CEA for 4 women.

In table 3 , there was no statistical difference related to surgical type, performed in women with benign and malignant tumors. Fertility-sparing surgery was the treatment of choice in 47 (73.4%) patients in benign germ cell tumor group and in 5 (71.4%) in malignant group. As related to sex cord-stromal tumors, 12 (35.3%) of the patients in the benign group and 11 (78.6%) of those in the malignant group were treated with fertility-sparing surgery. According to data verified on March 22, 2019 in the digital files of patients, the mean follow-up time of those 21 patients with malignant tumors was 44.2 months. Besides, one patient with germ cell tumor recurred and died of the disease, and one patient with sex cord-stromal tumor recurred.

**Table 3:** Women's treatment and follow-up

	**Germ cell tumor**			**Sex cord-stromal tumor**			
	**Benign n = 64**	**Malignant n = 7**	**P1**	**Benign n = 34**	**Malignant n = 14**	**P2**	**P3**
**Surgical type**							
Laparoscopy	28 (43.7)	2 (28.6)	0.595	10 (29.4)	3 (21.4)	0.4916	0.077
Laparotomy	36 (56.3)	5 (71.4)		24 (70.6)	11 (78.6)		
**Surgical treatment**							
Fertility sparing surgery	47 (73.4)	5 (71.4)	0.909	12 (35.3)	11 (78.6)	0.013	0.0034
HT + BSO/staging	17 (26.6)	2 (28.6)		22 (64.7)	3 (21.4)		
Chemotherapy							
Yes		5 (71.4)			1 (7.1)		
No		2 (28.6)			13 (92.9)		
Recurrence							
Yes		1 (14.3)			1 (7.1)		
No		6 (85.7)			13 (92.9)		

Abbreviations: HT + BSO, total hysterectomy and bilateral salpingo-oophorectomy; P1, including only germ cell tumors; P2, including only sex cord-stromal tumors; P3, including germ cell and sex cord-stromal tumors.

A 21-year-old patient with yolk sac tumor was submitted to unilateral salpingo-oophorectomy (tumor stage was Ic) and adjuvant chemotherapy. After 16 months, she presented serum α fetoprotein elevation and imaging exam showed bladder implant, perigastric, splenic and mesenteric lymph nodes disease. She was treated with chemotherapy, and since tumor presented partial response; she was submitted to laparotomy 29 months after the 1 ^st^ treatment. Biopsies were negative but after 5 months, imaging exam revealed intestinal disease progression. Once more, chemotherapy was used; however, the patient succumbed to the disease after 51 months from the diagnostic surgery.

A 53-year-old patient with gynandroblastoma was initially treated with total hysterectomy with bilateral salpingo-oophorectomy (HT + BSO) and pelvic and paraortic lymphadenectomy. Tumor stage was Ia. She recurred 19 months after it, and she was submitted to a laparotomy to resect a pelvic tumor (this time an adult granulosa cell tumor) and remained without disease until April 2017 when she was discharged from the hospital. Gynandroblastoma is currently classified as a sex cord-stromal tumor, not otherwise specified; which is a subtype of mixed sex cord-stromal tumor group.

## Discussion

In this single center study, we evaluated 119 benign and malignant non-epithelial ovarian tumors, as related to women's clinical features, preoperative CA125, HE4, and CEA serum levels, surgical and chemotherapy treatment, and disease recurrence. The majority of tumors were benign, mainly of germ cell origin (mature teratomas), and most of malignant tumors were diagnosed in the initial stage.

Among sex cord-stromal tumors, we detected a higher incidence of fibromas. Ovarian fibroma tumors account for ∼ 4% of all ovarian tumors. Fibromas can occur at any age, affecting adolescents and young women, although the mean age of occurrence is in the late forties. Interestingly, ∼ 10 to 15% of fibromas present with ascites, and less than 1% appear with both ascites and hydrothorax, known as Meigs syndrome, mimicking advanced ovarian cancer. 7 11 Fibromas usually present as solid adnexal mass on transvaginal ultrasound and, if CA125 is elevated, suspicion of malignancy increases. Shen et al showed that elevated serum CA125 level was found in 66 of 580 (11.3%) of patients with ovarian fibroma/fibrothecoma. Elevated serum CA125 level was significantly correlated with tumor diameter ≥ 10cm, ascites, and hydrothorax. 11

Women with benign tumors presented similar mean serum levels of CA125, HE4, and CEA when compared with women with malignant tumors. However, when we analyzed biomarker concentration by cutoff points, women with malignant germ cell tumors were significantly associated to elevated CA125, HE4, and CEA levels. Due to the rarity of non-epithelial ovarian cancer, there are limited data regarding the role of CA125, HE4, and CEA in the preoperative diagnosis of these rare tumors. In a previous study from our group, we found that women with non-epithelial ovarian cancer did not express elevated CA125 and HE4 levels such as women with epithelial ovarian cancer. 12

In our present study, fertility-sparing surgery was performed in 47 (73.4%) women with benign and in 5 (71.4%) women with malignant germ cell tumors. Preconized surgical treatment for young women is conservative (cystectomy or tumorectomy) with maintenance of ovarian parenchyma. Maintained cortical tissue contains follicles that are able to supply hormonal function and fertility. 13 In malignant germ cell tumors, conservative surgeries were performed, followed by adjuvant chemotherapy in 5 (71.4%) cases, with satisfactory response, except for the patient with yolk sac tumor. Fertility-sparing surgery was associated to chemotherapy in only one woman with malignant sex cord-stromal tumor, because of the poor response of this tumor to systemic therapeutics. 14 Recurrence was a relatively rare event for women with malignant non-epithelial ovarian tumors.

Surgeons should take into account the possibility of a synchronous or asynchronous bilateral benign ovarian tumor and focus on conservative surgery in young women. Although uncommon, even benign ovarian tumors can recur, for example, mature teratomas present ∼ 3 to 4% postsurgical recurrence. 15 On the other hand, postmenopausal women are safely treated with HT + BSO. 16

## Conclusion

In conclusion, mature teratomas were the germ cell ovarian tumors more frequently found in our casuistic. Among sex cord-stromal tumors, fibromas were the most common in our sample. Malignant cases were diagnosed at initial stages with good prognosis. Serum determination of CA125, HE4, and CEA levels were not useful for the preoperative diagnosis of malignancy in women with non-epithelial ovarian tumors.
